# Long-Term Physiological Alterations and Recovery in a Mouse Model of Separation Associated with Time-Restricted Feeding: A Tool to Study Anorexia Nervosa Related Consequences

**DOI:** 10.1371/journal.pone.0103775

**Published:** 2014-08-04

**Authors:** Sara Zgheib, Mathieu Méquinion, Stéphanie Lucas, Damien Leterme, Olfa Ghali, Virginie Tolle, Philippe Zizzari, Nicole Bellefontaine, Isabelle Legroux-Gérot, Pierre Hardouin, Odile Broux, Odile Viltart, Christophe Chauveau

**Affiliations:** 1 Université Lille Nord de France**,** Boulogne sur Mer, France; 2 Physiopathologie des Maladies Osseuses Inflammatoires, Boulogne sur Mer, France; 3 UMR INSERM 837, Développement et Plasticité du Cerveau Post-natal, Lille, France; 4 UMR-S 894 INSERM, Centre de Psychiatrie et Neurosciences, Université Paris Descartes, Sorbonne Paris Cité, Paris, France; 5 Service de Rhumatologie, Hôpital Roger Salengro, CHU Lille, France; 6 Université de Lille1, Villeneuve d’Ascq, France; Hosptial Infantil Universitario Niño Jesús, CIBEROBN, Spain

## Abstract

**Background:**

Anorexia nervosa is a primary psychiatric disorder, with non-negligible rates of mortality and morbidity. Some of the related alterations could participate in a vicious cycle limiting the recovery. Animal models mimicking various physiological alterations related to anorexia nervosa are necessary to provide better strategies of treatment.

**Aim:**

To explore physiological alterations and recovery in a long-term mouse model mimicking numerous consequences of severe anorexia nervosa.

**Methods:**

C57Bl/6 female mice were submitted to a separation-based anorexia protocol combining separation and time-restricted feeding for 10 weeks. Thereafter, mice were housed in standard conditions for 10 weeks. Body weight, food intake, body composition, plasma levels of leptin, adiponectin, IGF-1, blood levels of GH, reproductive function and glucose tolerance were followed. Gene expression of several markers of lipid and energy metabolism was assayed in adipose tissues.

**Results:**

Mimicking what is observed in anorexia nervosa patients, and despite a food intake close to that of control mice, separation-based anorexia mice displayed marked alterations in body weight, fat mass, lean mass, bone mass acquisition, reproductive function, GH/IGF-1 axis, and leptinemia. mRNA levels of markers of lipogenesis, lipolysis, and the brown-like adipocyte lineage in subcutaneous adipose tissue were also changed. All these alterations were corrected during the recovery phase, except for the hypoleptinemia that persisted despite the full recovery of fat mass.

**Conclusion:**

This study strongly supports the separation-based anorexia protocol as a valuable model of long-term negative energy balance state that closely mimics various symptoms observed in anorexia nervosa, including metabolic adaptations. Interestingly, during a recovery phase, mice showed a high capacity to normalize these parameters with the exception of plasma leptin levels. It will be interesting therefore to explore further the central and peripheral effects of the uncorrected hypoleptinemia during recovery from separation-based anorexia.

## Introduction

Chronic food restriction and the pathologic fear of weight gain are major symptoms described in restrictive-type anorexia nervosa (AN) patients. This disease mainly affects young girls with an average prevalence of 0.3% [1] and carries a high rate of morbidity, with osteoporosis being one of its major complications, occurring in 20–30% of cases depending on the studies [2], [3], and high fracture risk [4]. Nonetheless, biological analyses of patients do not reveal alterations of calcemia, phosphatemia and vitamin D level [5]. However, this psychiatric disease results in severe weight loss as shown by a mean body weight of 71% of that of healthy well-balanced weight controls, calculated from 10 different studies, and is frequently associated with chronic stress [6], [7]. The severity of the medical consequences is also linked to the duration of illness [8]. In particular, AN is associated with a nutritionally acquired resistance to growth hormone (GH), low leptinemia, high levels of adiponectin and cortisol, hypothalamic amenorrhea, osteopenia, and osteoporosis (reviewed in Méquinion et al [9]). At least some of these alterations are believed to be adaptive responses necessary to survive the severe and long-term caloric restriction. Nevertheless, a number of these physiological adaptations might be an obstacle for recovery [10] and could contribute to susceptibility to AN recurrence [11]. Most of the studies on key factors and mechanisms involved in the disease and on mechanisms related to the recovery are not possible in patients. Consequently, despite the combination of various and multidisciplinary therapeutic approaches, normalization of body weight and composition, and restoration of menses are hardly observed. Thus, valuable mouse models mirroring long-term alterations described in the disease and including a recovery phase are necessary.

An optimal model of AN should be developed in young females and be of sufficient duration for long-term adaptations to occur. Such a model should mirror the main alterations observed in patients, and particularly disturbance of body weight, body composition, plasma levels of adipokines, the GH/IGF-1 axis, the gonadotropic axis and energy metabolism. Ideally, it also should allow the follow-up of these alterations during a recovery phase.

Some attempts to develop animal models have been made to mimic and study AN consequences. The commonly used CR protocols (from 30% to 40%, which means 60 to 70% of *ad libitum* eaten) should be considered moderate, because they are determined from the average food intake of a control group fed *ad libitum* - which is classically 30% overfed taking into account its physiological needs [12]. Moreover, these restrictions without vitamin or mineral supplementation cause malnutrition in both mice and rats [13] that could, in turn, participate in the observed alterations usually attributed to lowered calorie intake [14]. However, these studies, that differed in age, sex, duration, percentage of restriction and food composition, showed that caloric restriction induces alterations of body composition, of various endocrine functions and of reproduction [15], [16], [17], [18].

Studies exploring severe food restriction are much less common. It has been shown that 50 to 70% food restriction [19], [20] includes a malnutrition proportional to the food restriction (reviewed in Cerqueira et al [13]). Moreover these last studies are of short duration while besides bone alterations, numerous changes in other tissues also need several weeks to develop [21].

Another kind of model mimicking AN alterations is based on voluntary activity in a wheel associated with a time-restricted feeding [22], [23]. These models were first supposed to induce a self semistarvation but later Boakes et al. demonstrated that this “starvation” was linked to dehydration [24]. This kind of model includes high physical activity levels that are also described in 31–80% of AN patients [25] and that impact on energy metabolism, reward circuitry and bone physiology. Thus, these models, are not representative of cases of AN with normal or low levels of physical activity which include the most severe cases.

Thus, a long-term mouse model combining most of the physiological alterations induced in severe restrictive AN patients and including the follow-up of a recovery phase is necessary to provide better strategies for disease management and treatment. In order to develop such a tool, we used a model of separation associated with time-restricted feeding partially characterized [26], [27]. The separation may induce physiological consequences linked to a stressful situation, thereby providing an animal model that offers the advantage that it includes chronic stress which is usually associated with AN. From a two-week study, authors pointed out the complementary and additive effects of the separation stress and the food restriction.

Here, the initial separation model was modified to rapidly induce a low body weight that could be maintained for a long period without malnutrition. This model is referred to as *Separation-Based Anorexia* (SBA) and has been especially characterized in regard to bone mass as well as hormonal and metabolic adaptations. To determine if some changes could definitively modify the phenotype of the restricted animals, a long-term protocol of recovery (REC) was also studied after the SBA phase.

The present study showed that SBA protocol induced severe and multiple alterations. We found noticeable physiological changes that mimicked those described in AN patients, particularly key endocrine adaptations and a stop of the bone mass acquisition. The recovery period revealed a high capability to correct most of these alterations including the low bone mass, but not the low leptin level.

## Materials and Methods

### Animals

Seven-week old female C57BL/6J mice (17–19 g) were purchased from Charles River Laboratories (St Germain sur l’Abresle, France). Mice were housed 6 per cage in a controlled room temperature (22°C±1°C) under a 12-hour dark/light cycle (lights off at 10 a.m.) with free access to water. The provided food was standard chow M20 at 2952.8 kcal/kg (Special Diets Services, St Gratien, France). Mice were acclimatized one week before the start of the protocol.

Ethics statement: Mouse care and treatment were conducted in accordance with institutional guidelines in compliance with national law and policy. This study was specifically approved by the Committee on the Ethics of Animal Experiments of Nord - Pas de Calais, France (Permit number: CEEA #022012).

### Short-term study

For the 2-week protocol, mice were randomly assigned to four different groups of 6 mice. The time-restricted feeding (TR) group was fed daily with an access to food gradually reduced from 6 h to 2 h a day along the protocol; the distribution of food was always done at the beginning of the dark phase. The separation (SEP) group was housed in a cage fitted with 6 individual Plexiglas partitions. The mice were able to smell and see each other without physical contact [27]. They were fed *ad libitum*. The SBA group was submitted to time-restricted feeding as described for the TR group and to separation as described for the SEP group. SBA mice were gathered together in regular cages for the periods of feeding. The control group (CT) was housed in standard conditions, with water and food *ad libitum*.

### Long-term study

**Figure 1:**
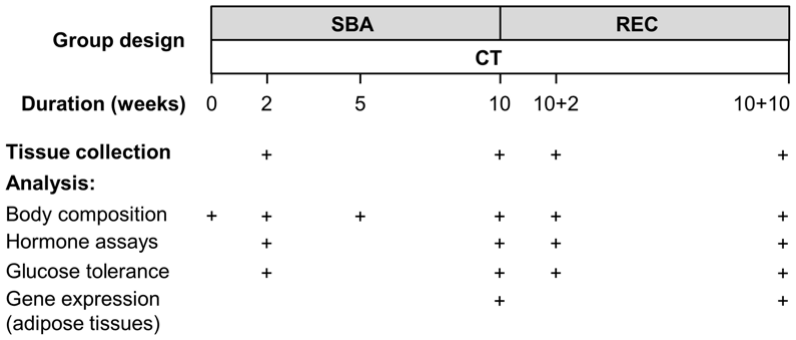
Design of the study. Forty mice were submitted to separation and time-restriction feeding (Separation-Based Anorexia, SBA). After 2 weeks, 10 mice were sacrificed. The others were kept in SBA conditions. 10 weeks after the beginning of the experiment, 10 mice were sacrificed and the 20 other mice were placed in standard conditions (Recovery, REC) during 2 or 10 more weeks, before sacrifice. Estrous cycles were followed all along the experiment. Forty other mice kept in standard housing conditions all along the experiment were studied and sacrificed according to the pattern used for SBA and REC mice.

The design of the mouse groups and the planning of analysis performed for the long-term study are shown in figure 1. For the long-term protocol, mice were randomly assigned to 8 different groups of 10 mice. SBA mice were submitted to SBA protocol, as described in the *short-term SBA protocol* section. REC mice were first submitted to a 10-week SBA protocol and thereafter to 2 or 10 weeks of recovery in standard housing conditions with food *ad libitum*. CT mice were kept in standard housing conditions for 2, 10, 12 or 20 weeks.

### Body Composition

Body composition was analyzed throughout the experiment in fasted and anesthetized mice between 09∶00 and 11∶00 by dual-energy X-ray absorptiometry (DEXA) using the Lunar PIXImus Mouse Densitometer (GE Healthcare, Madison, WI). Intramouse coefficients of variation were <5%.

### Intraperitoneal glucose tolerance testing (IPGTT)

To assess glucose tolerance, mice were fasted for 12 hr and i.p. injected with a glucose solution (1 g/kg) at the end of light phase. Their glycemia was assayed by using a glucose meter (Accu-Chek Performa glucometer, Roche, Rotkreuz, Switzerland) from blood sample drops with drawn at the tail just before and after 5, 10, 15, 30, 45, 70, and 90 min following glucose injection.

### Sacrifice

At different time points of the protocol (2, 10, 12 and 20 weeks), mice were sacrificed. All sacrifices were performed 7–8 hr after the beginning of the dark phase. Mice were fasted during 6 hr before anesthesia by pentobarbital (50 mg/kg). Glycemia was measured (Accu-Check Performa glucometer, Roche, Switzerland) at the same time. Blood was collected through cardiac puncture, immediately centrifuged (4000×g. for 10 min, 4°C) and serum aliquots were frozen in liquid nitrogen and stored (−80°C) until assayed. Tissue dissection and weighing included the right and left “triceps surae” hindlimb muscle group (including soleus and both lateral and medial heads of the gastrocnemius), inguinal and gluteal adipose tissue as subcutaneous adipose tissue (SCAT), periuterine adipose tissue as visceral adipose tissue (VAT) and interscapular brown adipose tissue (BAT). Tissues were immediately frozen in liquid nitrogen, before gene expression analysis. Ovaries were collected as mentioned below.

### Blood assays

All the samples were analyzed in duplicate. Plasma leptin levels were measured using Milliplex kit (Millipore, Billerica, USA) and the Luminex™ technology (Luminex Corporation, Austin, USA) to read the plates. Intra-assay coefficient of variation was <7% and inter-assay coefficient of variation was <23%. Plasma IGF-1 levels were determined with Quantikine Immunoassay kits (R&D Systems Inc., Minneapolis, USA). Whole blood growth hormone levels were measured with a sensitive sandwich ELISA adapted from Steyn et al [28]. Blood (4 µL) was collected from the tail vein always at the same period of the day and homogenized in 116 µL of 1X PBS-T buffer (0.05% Tween) and frozen at −20°C until GH assay. A monkey anti-rGH-IC-1 (AFP411S) was used as a capture antibody and a rabbit anti-rGH as detection antibody (AFP5672099) at a final dilution of 1∶40.000. Rat GH (rGH-RP2) was used as a standard. Standard and antibodies were provided by Dr Parlow (NIDDK-NHPP, Torrance, USA). Inter- and intra-assay coefficients of variations were <5%.

### Reproductive function

To assess reproductive function, vaginal smears were undertaken just before feeding. The tip of a pipette filled with saline solution (10 µl NaCl 9 g.l^−1^) was placed 5 mm into the vagina, flushed the vagina about 5 times and the final collect containing the vaginal secretion was put on glass slide. The cells were observed without coloration under light microscope Axio Skop (Zeiss, Oberkochen, Germany) equipped with a camera Digital Interface (Sony, Tokyo, Japan) with a final magnification of 100x [29]. After sacrifice, left and right ovaries were collected, fixed in 4% paraformaldehyde and then processed through graded alcohols into paraffin wax. Paraffin-embedded ovaries were serially sectioned at 5 µm thickness and stained with eosin/hematoxylin. Observations and photos were made using a Leica microscope (Wetzlar, Germany) equipped with a camera. Ovaries were measured following two axes (width and length) with Image J software (http://rsbweb.nih.gov/ij/).

### Gene expression analysis

Total RNAs were extracted from frozen SCAT and VAT using Extract-All (Eurobio, Les Ulis, France). Four micrograms were treated with DNase I (Roche Diagnostics, Penzberg, Germany) and reverse-transcribed using Maxima First Strand cDNA synthesis kit (Thermo Scientific, Waltham, USA) according to the manufacturer’s instructions. Real-time PCR analysis was performed using the LightCycler Nano instrument and the FastStart Essential DNA Green Master (Roche Diagnostics). Primers were designed using Oligo6 software and obtained from TIB MolBiol (Berlin, Germany). Selected primers exhibited a PCR efficiency included between 1.85 and 2 and sequences are available on request. Both cyclophilin A (PPIA) and Hypoxanthine-guanine phosphoribosyl transferase (HPRT) were used as internal controls to normalize gene expression. All results are expressed as fold-change compared to one SCAT of the CT group after 10 weeks of protocol.

### Statistical analysis

Values are presented as average ± SEM and statistics were generated by using GraphPad Prism (GraphPad Software Inc., San Diego, USA). The non-parametric Mann-Whitney U test was used to compare differences between two groups or between two durations within one group. Two-way ANOVA was used to test whether two regression lines represent independent populations, followed in some experiments by Bonferroni post-hoc test to compare differences between time matching points. All results were considered significant at *p*<0.05.

## Results

### Short term study: a rapid and severe weight loss induced by the combination of time-restricted feeding and separation

**Figure 2:**
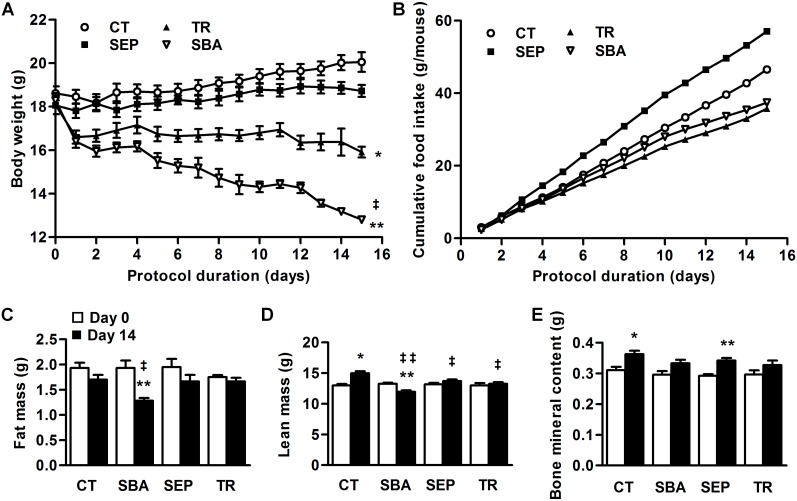
Weight, food intake and body composition of mice submitted to a 2-week study. Measures were performed on mice in standard conditions (CT), separated with food ad libitum (SEP), submitted to time-restricted feeding (TR) or separated and submitted to food access restriction (SBA). *A*: body weights were recorded daily before the eating period (beginning of the dark phase). *B*: Cumulative food intake was recorded for each group as the sum of the mean food intake per mouse from day 1 to day 15. *C–E*: Fat mass, lean mass and bone mineral content respectively were evaluated for each animal at day 0 and day 14, before food access. Data represent mean ± SEM; n = 6/group. In *A*, differences were tested by a 2-way Anova followed by a Bonferroni post-hoc test. SBA values are significantly different from CT values from day 1 to the end (**P<0.001). SBA values are significantly different from TR values from day 6 to the end *(* ^‡^ *P*<0.05). TR values are significantly different from CT values from day 1 to the end (*p<0.05). In *C*, *D*, and *E*, *p<0.05 and **p<0.005 when compared to day 0 of the same group; ^‡^ *p*<0.05 and ^‡‡^ *p*<0.005 when compared to CT group at the same duration.

To determine the impact of the combination of separation with time-restricted feeding (SBA), we compared body weight, body composition and food intake of four groups of mice submitted or not to separation and/or time-restricted feeding. This 2-week duration protocol allowed all SBA animals to reach the targeted body weight loss (25%) without showing signs of physiological distress. Indeed, the SBA group body weight showed the most severe decrease, reaching the targeted loss (*p*<0.005 *vs* day 0) (Fig. 2*A*), while its cumulative food intake was similar to the TR group (Fig. 2*B*). TR mice only showed a 12% decrease in body weight (*p*<0.05 *vs* day 0). On the contrary, the body weight of the separated (SEP) group remained stable over the 2 weeks protocol despite the highest cumulative food intake value. Finally, the control (CT) group exhibited a 7% increase of its body weight after the 2-week duration of the experiment (*p*<0.05 *vs* day 0).

The body composition of the 4 groups was determined before the beginning of the protocol (Fig. 2*C, D, E* *)*. After 2 weeks, SBA mice only showed a robust significant 33% decrease of the fat mass (*p*<0.005 *vs* day 0) and a modest but significant 9% decrease in the lean mass (*p*<0.005 *vs* day 0). The CT group showed only a significant increase in the lean mass (15%, *p*<0.001 *vs* day 0) while no significant change was noted for TR and SEP groups.

The bone mass of both CT and SEP mice showed a 17% increase (*p*<0.05 and *p*<0.005 respectively *vs* day 0), whereas TR and SBA mice gained 10% and 13% of bone mass respectively, without reaching statistical significance *vs* day 0.

The 2-week experiments pointed out the necessity to associate separation and restriction of food access to achieve rapidly the targeted weight loss, and this duration mainly altered the fat mass, and slightly impacted on the bone mass. Thus, SBA protocol was selected for long-term studies.

### Low body weight maintenance during the 10-week SBA protocol, but very fast recovery capacity

To study long-term physiological alterations and adaptations, SBA and REC mice were submitted to the SBA protocol for a 10-week period. Thereafter, mice of the REC group were housed again in standard conditions with food *ad libitum*, for up to 10 weeks of recovery protocol.

**Figure 3:**
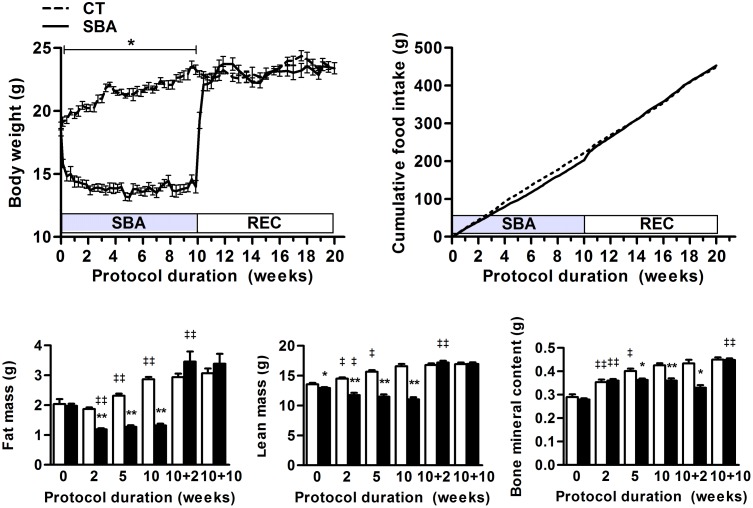
Weight, food intake and body composition of mice submitted to a 10-week SBA protocol followed by a 10-week recovery protocol (REC). Measures were performed on mice in standard conditions (CT) or separated and submitted to food access restriction (SBA). *A*: body weights were recorded before the eating period (beginning of the dark phase). *B*: Cumulative food intake was recorded for each group as the sum of the mean food intake per mouse from day 1 to day 140. *C–E*: Fat mass, lean mass and bone mineral content respectively were evaluated for each animal at the beginning and after 2, 5 and 10 weeks of SBA protocol or after 10 weeks of SBA protocol followed by 2 or 10 weeks of housing in standard conditions. Data represent mean ± SEM; n = 6–10/group. In *A*, differences were tested by a 2-way Anova followed by a Bonferroni post-hoc test. SBA values are significantly different from CT values from day 1 to day 70 (*p<0.0001). In *B*, *C*, *D* and E, *p<0.05, **p<0.005, when compared to corresponding CT value; ^‡^ *p*<0.05, ^‡‡^ *p*<0.005 when compared to the previous value of the same group.

During the long-term SBA protocol body weights were maintained about 25% under their initial weight, while CT mice continued to grow all along the protocol (Fig. 3*A*). After 10 weeks, the mean body weight of SBA mice was 40% under that of the matched CT group. Within 5 days of recovery, REC mice body weight reached the CT ones (Fig. 3*A*). The cumulative food intake of the SBA group reached 87% of that of the CT group after 10 weeks of SBA protocol (Fig. 3*B*). However, on the first day of the recovery period, REC mice began to eat more (8.15 g/day/mouse) than CT mice (3.21 g/day/mouse) and 3 to 4 days later their cumulative food became and remained similar to that of the CT group.

These data highlighted the specificity of SBA model, which associated a severe body weight loss with a slight underfeeding. Moreover, the REC phase showed the capability of mice to restore normal body weight and feeding behavior within few days.

### Low fat, lean and mineralized bone masses during the 10-week SBA protocol do not prevent the recovery of a normal body composition

**Figure 4:**
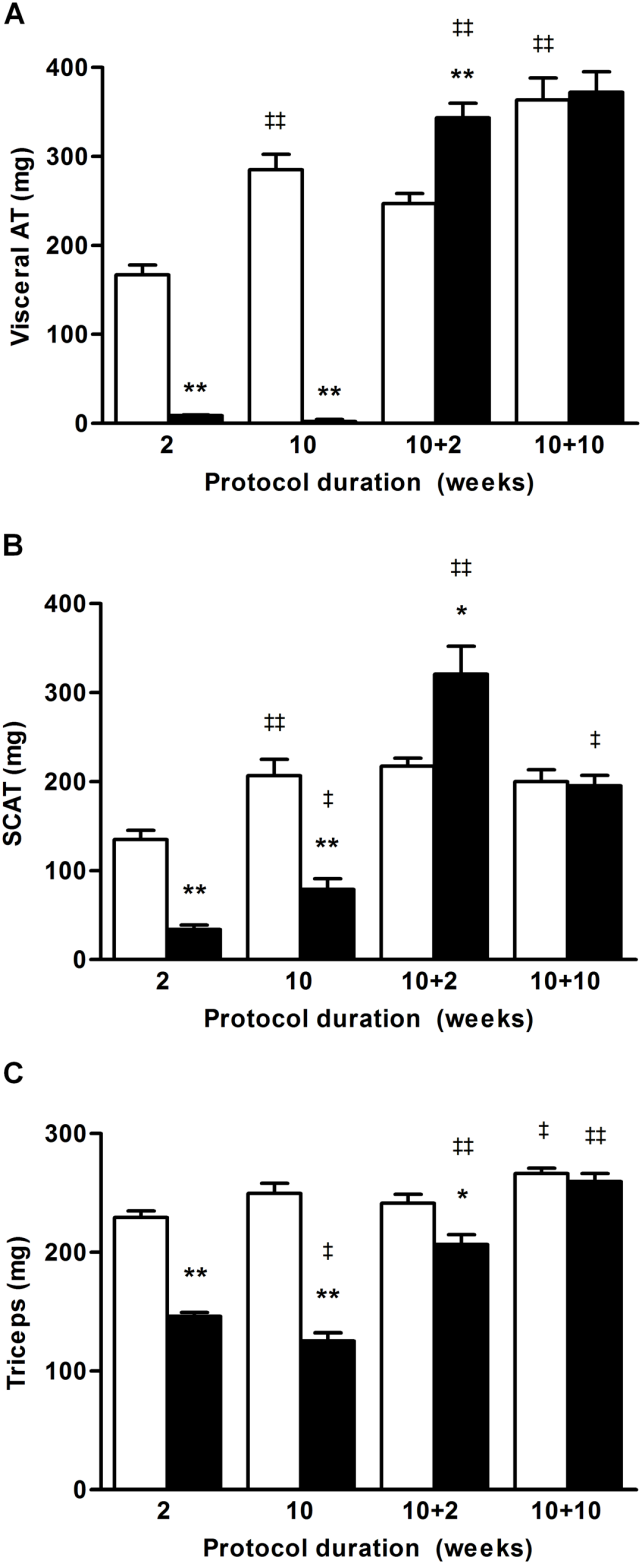
Weight evolution of visceral adipose tissue (AT), subcutaneous AT (SCAT) and triceps surae. Soft tissues from control and SBA mice were weighted after 2 or 10 weeks of protocol or after 10 weeks of SBA protocol followed by 2 or 10 weeks of housing in standard conditions. *A*: perigonadal fat was used to estimate the visceral fat mass evolution. *B*: SCAT, which gathers inguinal AT and AT around the leg, was used to estimate the sub-cutaneous fat mass evolution. *C*: Triceps surae were weighted to determine the muscle mass evolution. *p<0.05 and **p<0.005 when compared to corresponding CT group; ^‡^ *p*<0.05 and ^‡‡^ *p*<0.005 when compared to the previous value of the same group.

To determine the type of tissues participating to the body weight loss, we assessed the body composition of the CT and SBA groups (lean, fat and mineralized bone masses, respectively Fig. 3*C, D, E*) after 2, 5 and 10 weeks of SBA protocol, and after 10 weeks of SBA protocol followed by 2 and 10 weeks of REC protocol. The body weight increase of CT mice was related to an augmentation of fat, lean and bone mineral masses during the first 10 weeks of protocol. As previously shown in the short-term experiment, the present SBA protocol triggered a rapid 35% decrease of the fat mass (*p*<0.005 *vs* day 0, Fig. 3*C*) which was maintained during the 10-week protocol (Fig. 3*C*). These data were confirmed by a dramatic decrease in the weight of visceral (VAT) and subcutaneous (SCAT) adipose tissues (*p*<0.005 vs CT, Fig. 4*A*–*B*). As a consequence of having more metabolic activity, the perigonadal VAT was more depleted than the SCAT (loss of 99% *vs* 60% respectively). After 2 weeks of REC, whole body fat mass (*p*<0.005 *vs* week 10 of SBA, Fig. 3*C*), as well as VAT (*p*<0.005 *vs* CT and *p*<0.005 *vs* week 10 of SBA, Fig. 4*A*) and SCAT masses (*p*<0.05 *vs* CT and *p*<0.005 *vs* week 10 of SBA, Fig. 4*B*) rapidly increased. A complete normalization of these parameters was observed after 10 weeks of REC. Of note, interscapular brown adipose tissue (BAT) mass was slightly higher (+25%) in 2-weeks in SBA mice than in CT mice, and was normalized after 10 weeks. In 2-week REC mice, BAT mass was 45% higher than in CT mice and normalized after 10 weeks of REC protocol (data available as *Fig. S1*).

The lean mass of the SBA group decreased progressively reaching 89% of the day 0 value at the fifth week of protocol (*p*<0.005, Fig. 3*D*). We verified that this lean mass decrease included loss of muscular mass. Indeed, we showed a significant weight decrease of the triceps surae representative of skeleton muscles, (*p*<0. 005 SBA *vs* CT, Fig. 4*C*). After 2 weeks of REC, the lean mass increased (*p*<0.005 *vs* week 10 of SBA), reaching that of the CT value. However, even if the weight of the triceps surae increased after 2 weeks of REC (*p*<0.005 *vs* week 10 of SBA, Fig. 4*C*) it remained low compared to the CT group and was fully normalized at 10 weeks of REC.

Finally, SBA mice presented a delay in the acquisition of bone mass, compared to the CT group (Fig. 3*E*). Indeed, no statistical changes were noted for values of SBA mice after 2, 5 and 10 weeks of protocol. This could suggest that the bone mass gain was ended after the second week of SBA protocol. Interestingly, the bone mass of the REC group remained significantly lower than the CT group (*p*<0.05) within the first 2 weeks of REC. It increased later to reach that of the CT group at the end of the protocol (Fig. 3*E*).

Thus, the long-term SBA protocol induced a significant blockade of the bone mass acquisition and all the alterations were fully normalized within the 10 weeks of the REC protocol.

### Hypoleptinemia during long-term SBA protocol is incompletely corrected after long-term recovery

**Figure 5:**
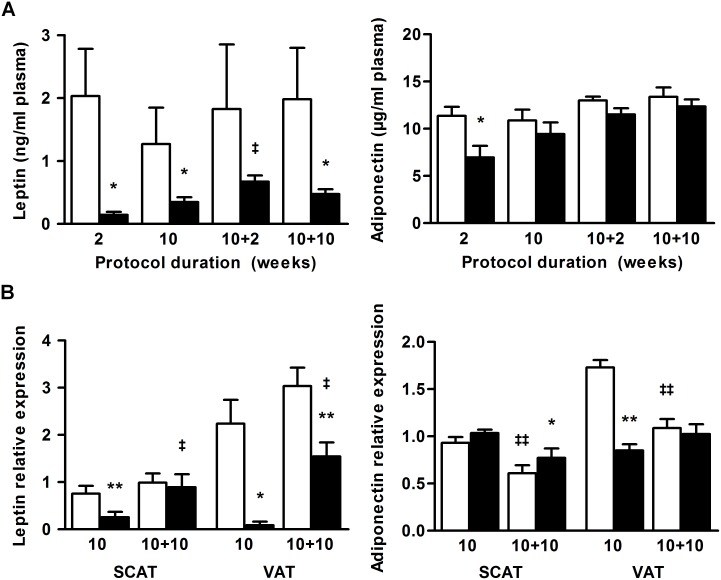
Leptin and adiponectin. *A*: Plasma concentrations of leptin and adiponectin of mice in standard conditions, CT(□), or separated and submitted to food access restriction, SBA(▪) after 2 and 10 weeks of protocol, followed by 2 and 10 weeks of standard housing conditions. *B*: Relative leptin and adiponectin mRNA levels in SCAT and VAT vs HPRT and PPIA housekeeping genes. Data represent mean ± SEM; n = 6–10/group. *p<0.05 and **p<0.005 when compared to CT group at the same duration; ^‡^ *p*<0.05 and ^‡‡^ *p*<0.05 when compared to the previous value of the same group.

Because hypoleptinemia is one of the main endocrine dysregulation in AN patients and due to its involvement particularly in the regulations of food intake, energy metabolism and bone mass, we assayed plasma leptin levels. Plasma adiponectin concentrations were also analyzed since studies on AN patients showed contradictory results for this adipokine that has an important role in metabolic regulation. In accordance with the decrease in fat mass, plasma levels of leptin were drastically decreased in the SBA group compared to control after 2 and 10 weeks of the protocol (*p*<0.05, Fig. 5*A*). After 10 weeks of the REC protocol, leptinemia remained low despite a totally normalized fat mass (*p*<0.05, Fig. 5*A*). This surprising result was confirmed by the reduced leptin mRNA level in adipose tissues (Fig. 5*B*). Indeed, in VAT, the main adipose tissue secreting leptin, its expression level only increased to reach 50% of that of the CT group (*p*<0.005) after recovery. However, in SCAT, which is a more modest source of leptin, its mRNA levels were normalized. Contrastingly, plasma adiponectin levels appeared to be significantly lower in SBA mice only after 2 weeks of SBA protocol, while adiponectin mRNA levels were significantly lower in VAT of 10-week SBA mice only (Fig. 5*A*).

Thus, SBA protocol induced a strong hypoleptinemia that was only slightly reversed during REC protocol, despite a normalized fat mass.

### Reversible alteration of GH/IGF-1 axis during long-term SBA protocol

**Figure 6:**
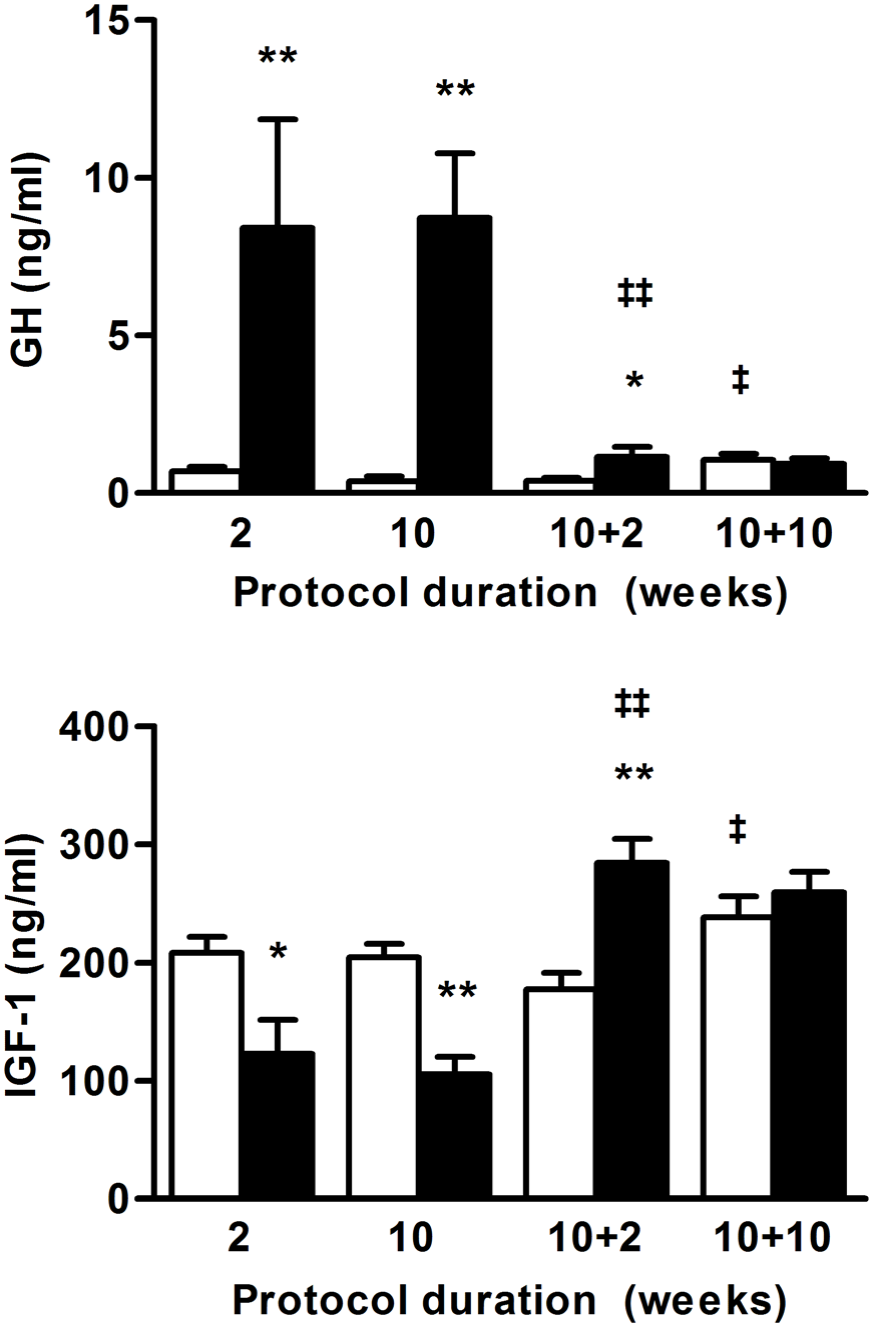
GH and IGF-1. Whole blood GH levels and plasma IGF-1 levels were assayed on mice in standard conditions (CT), or separated and submitted to food access restriction (SBA) after 2 and 10 weeks of protocol, followed by 2 and 10 weeks of standard housing conditions. Data represent mean ± SEM; n = 6–10/group. *p<0.05 and **p<0.005 when compared to CT group at the same duration; ^‡^ *p*<0.05 and ^‡‡^ *p*<0.005 when compared to the previous value of the same group.

AN patients exhibits high plasma GH levels and low plasma IGF-I levels leading to hypothesize a nutritionally mediated and acquired resistance to GH [30]. As shown in Fig. 6, such results were also obtained with the SBA protocol. Indeed, 2-week and 10-week SBA protocols induced a 10 fold increase in blood GH concentrations (*p*<0.005 vs CT). This GH increase was associated with nearly 2-fold lower concentrations of plasma IGF-1 (*p*<0.05 and *p*<0.005 after 2 and 10 weeks respectively). In the REC period, blood GH levels decreased quickly and were fully corrected after 10 weeks. Plasma IGF-1 levels increased over that of CT mice after 2 weeks of REC (*p*<0.005), before normalization.

These results suggest a potential liver resistance to GH, as described in mice after short-term severe food restriction [31], [32] and AN patients.

### High and reversible increase in glucose clearance

**Figure 7:**
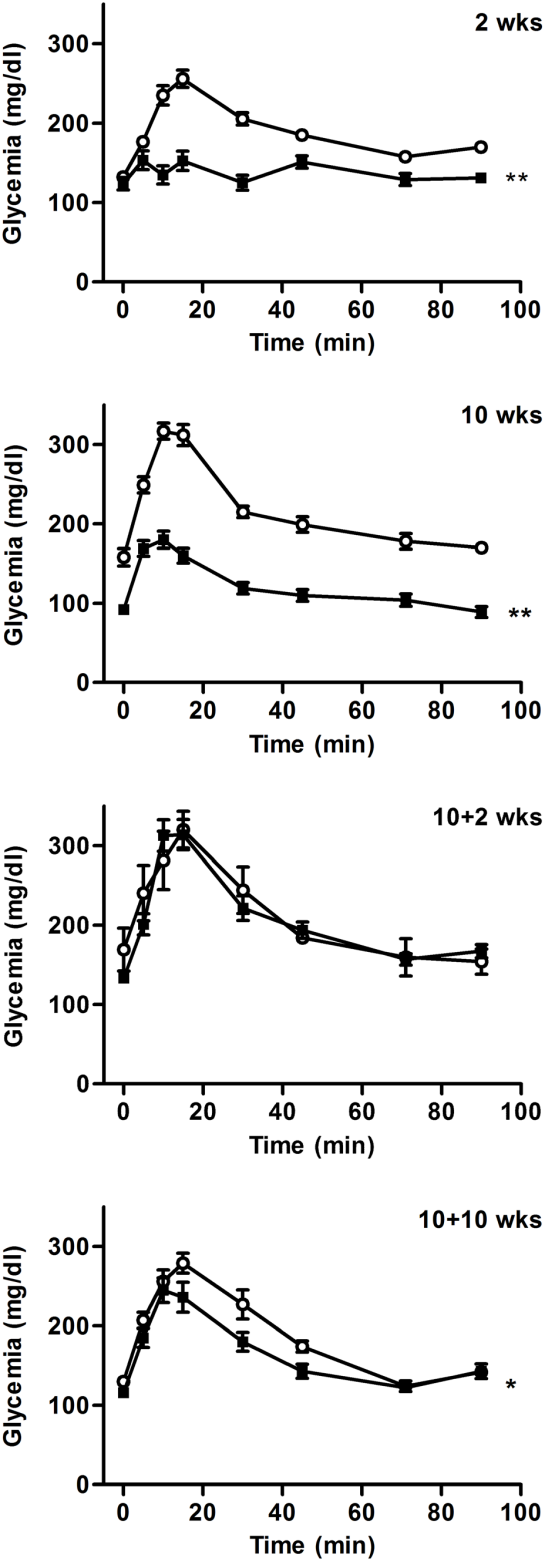
Intraperitoneal glucose tolerance test in mice in standard conditions (CT), or separated and submitted to time-restricted feeding (SBA) after 2 and 10 weeks of protocol, followed by 2 and 10 weeks of standard housing conditions. Data represent mean ± SEM; n = 6–10/group. *p<0.05 and **p<0.0001 significant differences between the two curves using Two-way ANOVA.

Considering the profound alterations in lean and fat mass, whole glucose homeostasis was analyzed at the different steps of the protocol using intraperitoneal glucose tolerance tests in overnight fasted mice (Fig. 7). After 2 weeks of protocol, SBA and CT mice displayed similar glycaemia. After glucose injection, SBA mice failed to increase their glycaemia, which suggests a very high capability to rapidly clear the glucose in comparison to CT mice (*p*<0.0001). After 10 weeks of SBA protocol, this clearance appeared less efficient than at week 2, but remained faster than in the CT group (*p*<0.0001). Finally, after 2 weeks of REC clearance capacities were the same in REC and CT, while after 10 weeks, REC mice showed a slightly faster clearance than CT mice (*p*<0.05).

We concluded that the SBA protocol triggered an enhanced, and yet reversible, glucose disposal which is reminiscent of the improved glucose homeostasis with enhanced insulin sensibility shown in rodent models of caloric restriction [33], [34].

### SBA protocol induced severe but reversible changes in reproductive function

**Figure 8:**
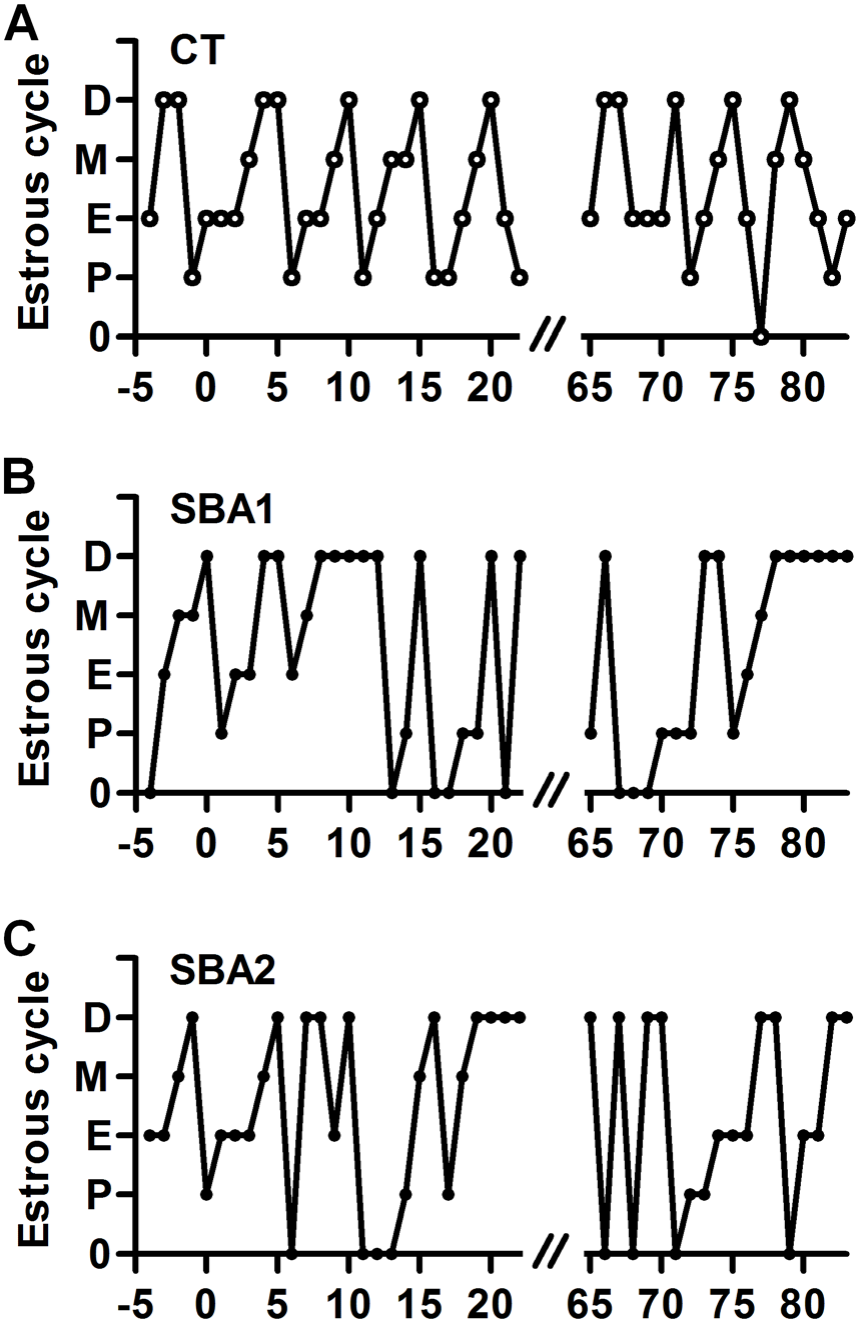
Estrous cycle alteration. Estrous cycle determined according to the observation of the cell population of vaginal washes was daily followed on mice in standard conditions (CT), or separated and submitted to food access restriction (SBA) from day 0 to day 70, followed by 20 days of standard housing conditions. D = diestrus, M = metestrus, E = estrus, P = proestrus, 0 =  no cell observed. *A*: A representative example of estrous cycle of CT mice. *B*: A representative example of cycles observed in SBA mice, with the onset of long duration diestrus during the recovery period. *C*: A representative example of cycles observed in SBA mice, with the onset of estrus during the recovery period.

**Figure 9:**
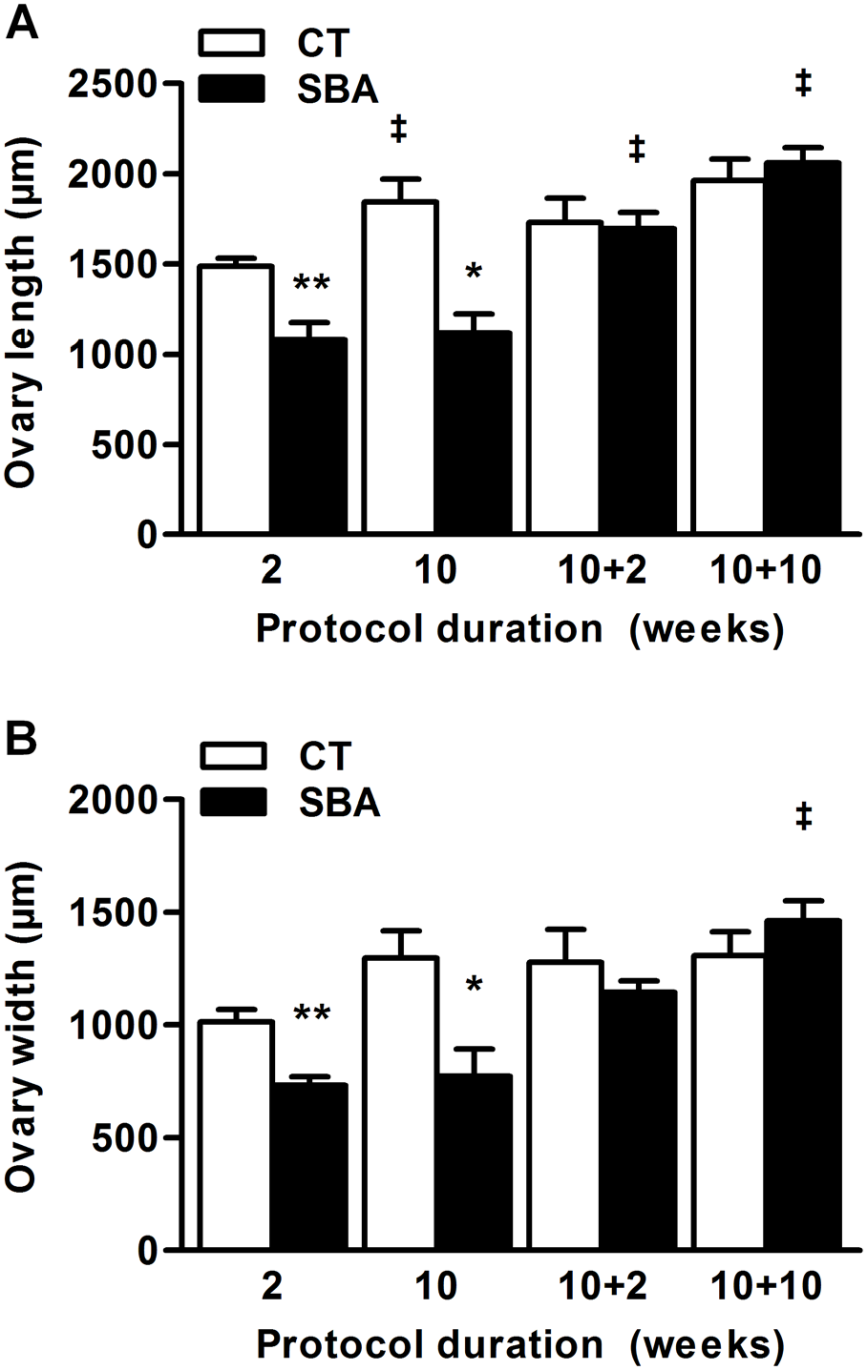
Alterations of reproduction. Ovary size of mice in standard conditions (CT), or separated and submitted to food access restriction (SBA) after 2 and 10 weeks of protocol, followed by 2 and 10 weeks of standard housing conditions. *A*: Ovary length measured on ovary slices. *B*: Ovary width measured on ovary slices. Data represent mean ± SEM; n = 6/group. *p<0.05 and **p<0.005 when compared with CT group at the same duration; ^‡^ *p*<0.05 when compared with previous value of the same group.

Linked to low fat mass and low leptinemia, most of the AN patients are amenorrheic. Similarly, reproductive function appeared to be altered very quickly in the SBA mice, as shown by the decrease of estrus frequency (Fig. 8) and by atrophy of the ovaries (Fig. 9, *p*<0.005 after 2 weeks of protocol, *p*<0.05 after 10 weeks *vs* CT group). Two weeks of REC protocol were sufficient to restore a normal ovary size. Estrus cycle recovery was more heterogeneous during the first 2 weeks. Indeed, some REC mice returned to normal cycles, while others showed long duration diestrus without estrus phase, before normalization within 10 weeks of REC.

Thus, the SBA protocol appeared to mimic the alterations in reproductive function observed in AN patients and calorie restricted rodents [17].

### Increased mRNA levels of genes involved in lipogenesis, fatty acid oxidation and brown adipocyte phenotype in WAT

**Figure 10:**
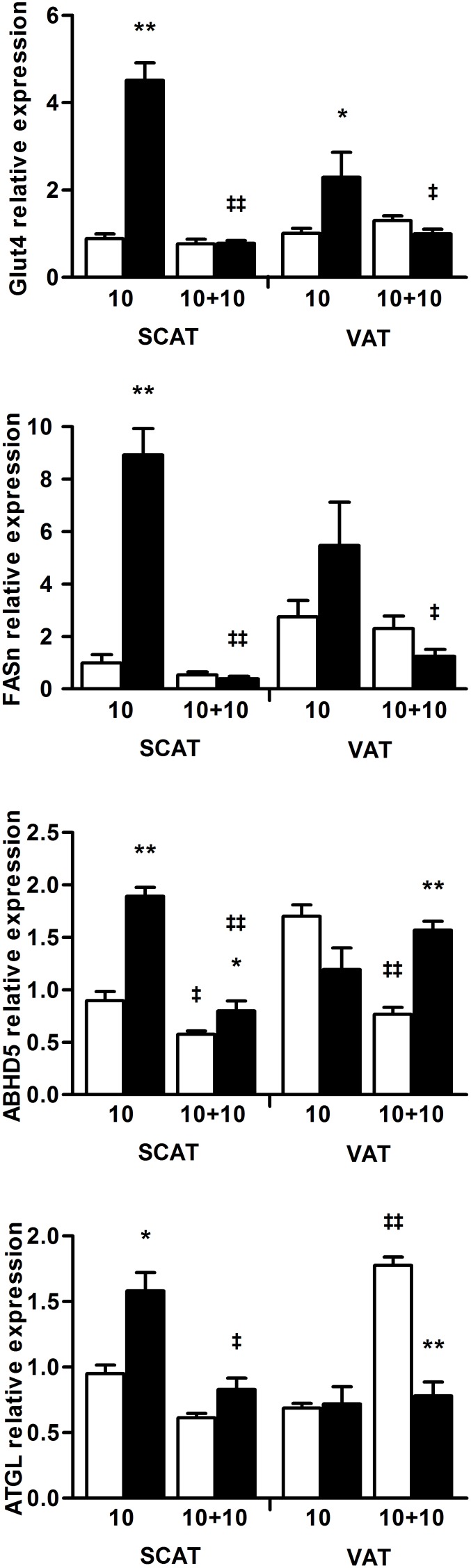
Expression analysis in adipose tissues of genes involved in lipid metabolism. Relative mRNA levels of Glut4, FASn, ABHD5 and ATGL were determined by real-time PCR experiments, in subcutaneous (SCAT) and visceral adipose tissues (VAT) of control □ and SBA ▪ mice. PPIA and HPRT were used as housekeeping genes. All results are expressed as fold-change compared to one SCAT of the control group after 10 weeks. Analyses were done after 10 weeks of SBA protocol and 10 additional weeks of REC protocol. Data represent mean ± SEM; n = 5–10/group. *p<0.05 and **p<0.005 when compared to CT group at the same duration; ^‡^ *p*<0.05 and ^‡ ‡^ *p*<0.005 when compared to the previous value of the same group.

The maintenance of a low fat mass despite an almost unaltered food intake during the SBA protocol pointed out a potential unbalance in energy metabolism induced by the chronic stress. To determine some of these metabolic adaptations, inguinal SCAT and periuterine VAT were further analyzed using real-time PCR analysis. The 10-week SBA protocol induced an increase in the mRNA level of the glucose transporter Glut-4 (by more than 4-fold) and the lipogenic enzyme FASn (Fatty Acid Synthase, by more than 8 folds) compared to the CT group (Fig. 10). This effect, reflecting a potential increase in fatty acid synthesis, was more pronounced in the SCAT than in the VAT, as already shown [35]. Regarding genes involved in lipolysis, the Acyl triglyceride lipase (ATGL) and its limiting cofactor ABHD5/CGI-58 mRNAs were only higher (1.5-fold and 2-fold, respectively) in the SCAT after the prolonged SBA protocol (Fig. 10). The expression of lipolytic genes was unaltered in VAT in accordance with Higami et al [36] and with their predominant post-translational regulation.

**Figure 11:**
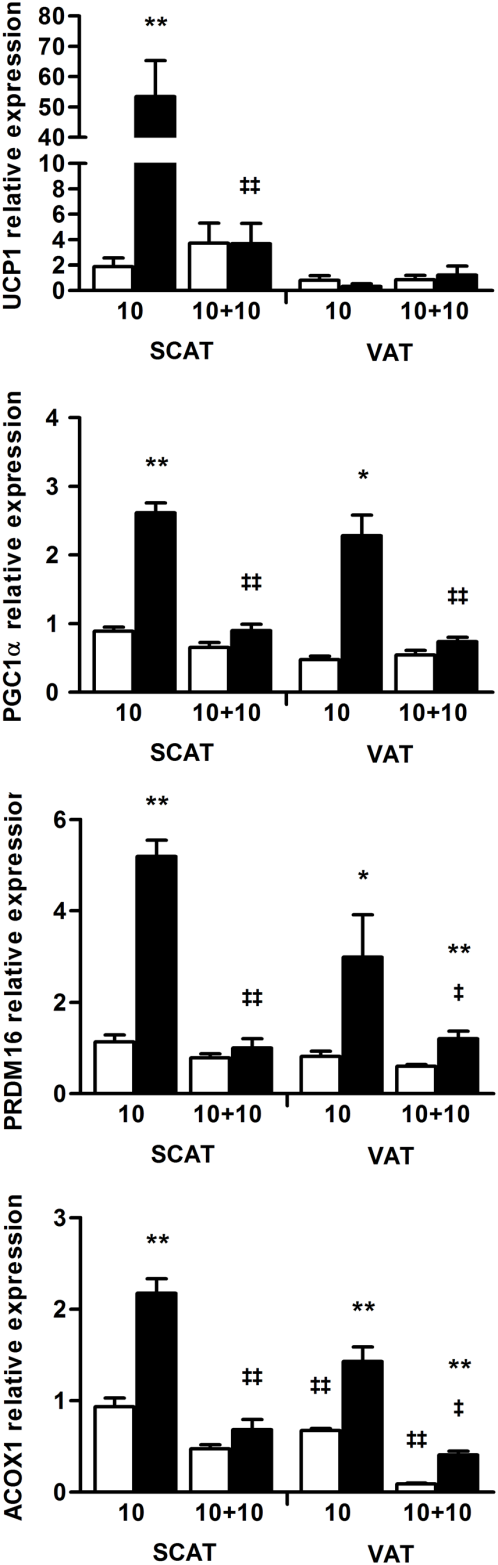
Expression analysis in adipose tissues of genes involved in brown adipocyte phenotype. Relative mRNA levels of UCP1, PGC1α, PRDM16 and ACOX1 were determined by real-time PCR experiments, in subcutaneous (SCAT) and visceral adipose tissues (VAT) of control □ and SBA ▪ mice. PPIA and HPRT were used as housekeeping genes. All results are expressed as fold-change compared to one SCAT of the control group after 10 weeks. Analyses were done after 10 weeks of SBA protocol and 10 additional weeks of REC protocol. Data represent mean ± SEM; n = 5–10/group. *p<0.05 and **p<0.005 when compared to CT group at the same duration; ^‡^ *p*<0.05 and ^‡ ‡^ *p*<0.005 when compared to the previous value of the same group.

Moreover, long-term caloric restriction in rodents is expected to shift metabolism toward fatty acid oxidation [35] and to promote mitochondrial biogenesis [36], [37] in white adipose tissues. The mRNA levels of the transcriptional coactivator PGC1α, involved in mitochondriogenesis, of the key transcriptional regulator of the brown adipocyte lineage Prdm16 and of the peroxisomal acyl-coenzyme A oxidase 1 (ACOX1), an enzyme involved in fatty acid beta-oxidation, were all significantly increased in the VAT and the SCAT of mice subjected to the prolonged SBA protocol. Interestingly, in agreement with its role in driving the brown adipocyte gene program specifically in SCAT, the higher level of Prdm16 mRNA was associated with a drastic up-regulation of the uncoupling protein UCP1 mRNA which was 25 times more expressed in the SCAT of the SBA mice compared to CT mice (Fig. 11).

Our data highlighted that white adipose tissues adapted their lipid metabolism during the prolonged SBA protocol in a similar way to animal models of negative energy balance such as long-term caloric restriction. Furthermore, the development of brite/beige adipocytes in the SCAT was supported by the up-regulation of several critical genes and could indicate a rise in thermogenesis. Finally, most of the gene expression alterations were reversed after 10 weeks of the REC protocol. Of note, Glut4 and UCP1 mRNA levels were not altered in the BAT of SBA and REC mice (data available as *Fig. S2*).

## Discussion

To develop an AN model mimicking early and late physiological consequences of severe AN, we sought to characterize the long-term physiological alterations induced by chronic stress associated with time-restricted feeding. The long-term recovery capabilities were also determined by examining alterations potentially involved in this process.

Time-restricted feeding was chosen, as it permits food intake close to that of *ad libitum* mice and thus facilitates survival. Separation was used as a cause of chronic stress to both induce a severe body weight decrease and to enrich the model with a factor potentially involved in some alterations related to AN. Two studies partly described A model combining time-restricted feeding and separation was partly described in two studies [26], [27]. We adapted this model to young C57BL/6 female mice, and referred to it by the name of separation-based anorexia (SBA). The proposed specifications were related to daily food consumption close to that of *ad libitum* group, a 25% body weight loss within the 2 first weeks, the maintenance of this low level for 8 more weeks and significant impact on bone mass.

First, this study showed that the combination of time-restricted feeding and separation was necessary to induce a fast body weight decrease of at least 25% *vs* initial weight, similar to that observed in AN patients. Second, this SBA protocol allowed us to maintain the mice at this low body weight for up to the end of the 10-week protocol. The body weight loss was linked to a marked lowering of fat and lean mass and to termination of bone mass acquisition. In accordance with their low fat mass, SBA mice exhibited hypogonadism, alterations of key endocrine parameters (hypoleptinemia, modifications in the GH/IGF-1 axis). Altogether these data demonstrate that the SBA protocol induces physiological alterations similar to those observed in AN patients. These data validate the SBA as a valuable model to study some of the main physiological alterations described in AN.

The fat depletion triggered by the SBA protocol was puzzling with regard to the unchanged food intake. Time-restricted feeding, when applied during the dark phase, has recently been shown to moderately lower body weight and to modify the time frame of energy expenditure and fuel utilization without affecting food intake [38], [39]. Indeed, the initial characterization of TR mice during 2 weeks showed that, despite a similar food intake, the protocol led to a significant decrease in body weight albeit with minor alterations in the whole body composition. When applied alone, separation resulted in higher food consumption with similar body weight gain in SEP mice compared to CT ones. This could indicate that the separation protocol may increase energy expenditure, either *via* chronic stress-induced stimuli or *via* increased needs of thermogenesis (the mouse is alone in its box). Combination of both time-restricted feeding and separation was required to obtain a severe decline in body weight and fat mass without markedly affecting food consumption. These first observations suggest that the energetic balance is modified in our SBA model. We acknowledge that the involvement of disturbed nutrient digestion and absorption associated with the time-restricted feeding schedule cannot be discarded; however such hypothesis seems unlikely to explain the severe energy imbalance observed in the SBA group. An increase in physical activity, like anticipatory activity before food intake, does not seem able to impact so negatively on the energy balance. Moreover it should have impacted the TR group to a similar extent, and this was not observed.

To delineate the SBA-induced adaptations, the mRNA level of critical metabolic genes was measured in SCAT and VAT after 10 weeks of protocol. The gene expression changes corresponded to those described in perigonadal adipose tissue following long-term caloric restriction [35], [36], [37] and could support a shift of adipocyte metabolism toward higher lipogenesis and fatty acid oxidation capacities [35]. Importantly, several genes (UCP1, PGC1α, Prdm16) were up-regulated in the SCAT of SBA mice suggesting the emergence of beige/brite adipocytes in this specific fat depot. Such a potential “britening” of the SCAT may be caused by greater needs in thermogenesis due to the separation of the mice. Indeed, the appearance of beige/brite adipocytes has been observed in many species after cold exposure but also treatment with beta 3-adrenergic agonists [40], [41]. To note, the development of brite thermogenic adipocytes remains poorly investigated in long-term caloric restriction animals despite the report of decreased body temperature [42]. For example, an enhancement of UCP1 expression in inguinal fat has been reported in the Lou/C rat [43], a model of spontaneous food restriction with high energy expenditure and increased sympathetic activity in adipose tissues. Rogers et al [44] described the effects of long-term caloric restriction (60% eaten) on SCAT “britening” with higher levels of UCP1. Their study also pointed out the potential involvement of adrenergic tone decrease and disappearance of brown-like adipocytes in SCAT with aging. In SBA mice, the increased need of thermogenesis and the chronic stress, both separation-induced, could be responsible for a higher adrenergic tone and thus an enhancement of SCAT “britening”.

Taken together, our data on the SBA protocol strongly suggest that it induced an increase in energy demand leading to a wide metabolic adaptation.

The recovery protocol revealed a high capacity of mice to correct the numerous and substantial alterations that occurred during the long-term SBA phase. Interestingly, this included the normalization of bone mass when compared to age-matched CT mice. Because most of the AN recovered patients keep a low bone mass, understanding the mechanisms allowing its normalization in SBA mice could be of importance for the development of new options of treatment of this AN-specific osteoporosis. Alterations potentially involved in the low bone mass of patients - low circulating IGF-1, low leptinemia, disruption of ovarian functions leading to estrogen level decrease - (Reviewed in Méquinion et al [9]) are reproduced in SBA mice, and thus should also be involved in their bone mass alteration. Consequently, this model could be useful to determine which alterations should be corrected to reduce the bone loss. In the REC phase, the main difference found for these factors between SBA mice and recovered patients is the persisting low plasma leptin level despite a fully normalized fat mass in REC mice. In studies investigating AN patients, short-term weight gain seems to induce an increase in leptinemia. This leptinemia when adjusted for BMI and % body fat was higher than in healthy controls but uncorrected leptinemia remained lower or equal to that of controls [45], [46]. Patient’s leptinemia was found to be normalized when recovery is maintained in the long term [47]. In SBA mice, it could be thought that leptinemia was corrected before the end of the first 2 weeks of REC, but the absence of later normalization does not support this hypothesis. Thus, the full bone mass recovery and the persisting hypoleptinemia after the REC protocol revealed major differences with recovered AN patients. On the one hand this is a failure in mimicking the AN recovery process, but on the other hand these differences pointed out the potential key role of leptin level in the recovery process.

Indeed, in the SBA model hypoleptinemia could be involved in the fast body fat mass normalization, as it could participate in keeping a low level of energy expenditure. In this unique context, low leptinemia could also induce a reduced stimulation of the sympathetic nervous system and thus improve bone mass acquisition which is supported by the normalized GH/IGF-1 axis and ovary activity. Indeed, in mice CR-induced decrease in bone mass is prevented by propranolol (a beta-blocker), whereas isoproterenol (a beta-stimulant) reduces bone volume in CT mice [16]. The involvement of hypoleptinemia in the SBA mouse recovery remains to be tested in experiments including leptin treatments during the REC phase.

The key role of leptin is also suggested in the recovery of patients, as high leptin levels subsequent to weight gain were suggested to be the cause of increased energy expenditure during this stage of disorder and were found to predict renewed weight loss [48].

Other questions also remain to be answered. Indeed, it would be useful to determine if, like for AN patients [5], SBA protocol effects on bone mass and microarchitecture are site-dependent. Previous studies showed the importance of local IGF-1 production for bone physiology, and it will be interesting, therefore, to determine if the GH resistance often described in the liver also takes place in SBA mouse bones. From a metabolic and neurobiological point of view, it will be of interest to determine how the brain decodes the low leptin level in mice with normalized fat mass. Is there a central nervous system recalibration allowing a signaling corresponding to a normal fat mass or does the brain still integrate the low leptin level as a signal of a low fat mass?

In summary, the present study strongly supports SBA as a valuable model of prolonged state of negative energy balance which mimics numerous symptoms observed in AN. It shows that SBA model could be useful to study different hypothesis regarding the involvement of each described alterations in the medical consequences of AN. Following the SBA protocol, the recovery phase revealed a high capacity of mice to normalize the long-term alterations. It also pointed out, however, the uncorrected low leptin levels, despite a fully recovered fat mass. The consequences of this persisting hypoleptinemia on the recovery process remain to be determined.

## Supporting Information

Figure S1 **Weight evolution of brown adipose tissue.** Interscapular brown adipose tissue (BAT) from control □ and SBA ▪ mice were weighted after 2 or 10 weeks of protocol followed by 2 or 10 weeks of housing in standard conditions. Data represent mean ± SEM; n = 4–6/group. * *p*<0.05 when compared to corresponding CT group.
(TIF)

Figure S2 **Gene expression analysis in brown adipose tissue.** Relative mRNA levels of Glut4 and UCP1 were determined by real-time PCR experiments, in brown adipose tissue (BAT) of control □ and SBA ▪ mice. PPIA and HPRT were used as housekeeping genes. All results are expressed as fold-change compared to one subcutaneous adipose tissue of the control group after 10 weeks of protocol. Analyses were done after 10 weeks of SBA protocol and after 10 more weeks of REC protocol. Data represent mean ± SEM; n = 5–10/group. No significant difference was found.
(TIF)
