# Supplementary figures and images for: Long-Term Physiological Alterations and Recovery in a Mouse Model of Separation Associated with Time-Restricted Feeding: A Tool to Study Anorexia Nervosa Related Consequences
Source: PLoS One. 2014 Aug 4;9(8):e103775. doi: 10.1371/journal.pone.0103775 (PMC4121212; doi:10.1371/journal.pone.0103775)

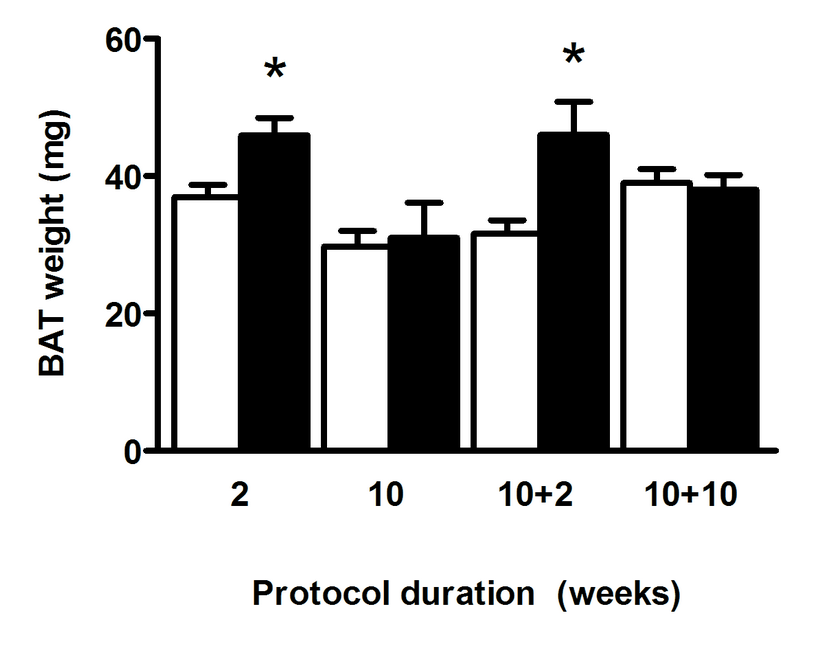

Supplement: Figure S1 — Weight evolution of brown adipose tissue. Interscapular brown adipose tissue (BAT) from control □ and SBA ▪ mice were weighted after 2 or 10 weeks of protocol followed by 2 or 10 weeks of housing in standard conditions. Data represent mean ± SEM; n = 4–6/group. * p<0.05 when compared to corresponding CT group. (TIF) [file pone.0103775.s001.tif]

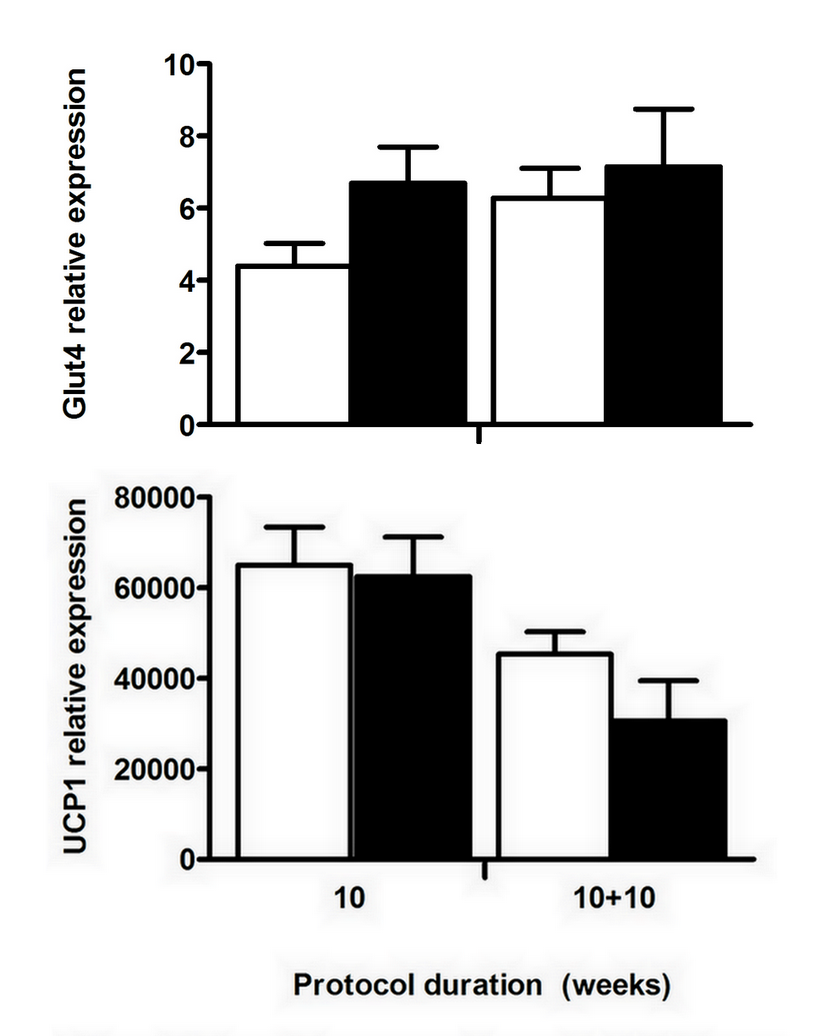

Supplement: Figure S2 — Gene expression analysis in brown adipose tissue. Relative mRNA levels of Glut4 and UCP1 were determined by real-time PCR experiments, in brown adipose tissue (BAT) of control □ and SBA ▪ mice. PPIA and HPRT were used as housekeeping genes. All results are expressed as fold-change compared to one subcutaneous adipose tissue of the control group after 10 weeks of protocol. Analyses were done after 10 weeks of SBA protocol and after 10 more weeks of REC protocol. Data represent mean ± SEM; n = 5–10/group. No significant difference was found. (TIF) [file pone.0103775.s002.tif]
